# Earthworms With Low Dose n‐MoS_2_ Improve Soybean Yield and Quality by Reconfiguring Rhizosphere Chemistry and Strengthening Nitrogen Fixation

**DOI:** 10.1111/ppl.70988

**Published:** 2026-06-28

**Authors:** Muhammad Nadeem, Muhammad Adeel, Usama Zaheer, Noman Shakoor, Imran Azeem, Taiming Zhang, Yukui Rui

**Affiliations:** ^1^ Beijing Key Laboratory of Farmland Soil Pollution Prevention and Remediation and College of Resources and Environmental Sciences China Agricultural University Beijing China; ^2^ Beijing Normal University at Zhuhai Zhuhai Guangdong China; ^3^ Department of Environmental Engineering Izmir Institute of Technology Izmir Turkey; ^4^ College of Life Sciences and Oceanography, Shenzhen University Shenzhen China; ^5^ School of Breeding and Multiplication (Sanya Institute of Breeding and Multiplication), Hainan University Sanya China

**Keywords:** bioavailability, earthworm, nanofertilizer, rhizosphere chemistry, seed quality

## Abstract

Nano‐enabled fertilizers can enhance biological nitrogen fixation (BNF) and yield in legumes; yet, their interactions with earthworms remain poorly understood. Herein, we performed a 120‐day greenhouse experiment applying low‐dose n‐MoS_2_ at 10 mg kg^−1^ with earthworms to evaluate interactive effects on soybean yield and underlying mechanisms. Earthworms combined with n‐MoS_2_ treatment promoted Mo release and transport, with translocation and bioaccumulation increased by 44% and 26%, respectively. Furthermore, the rhizosphere soil shifted towards NO_3_^−^ dominance while pH remained stable and consistent with sustained nitrification. Belowground, root system architecture expanded by 33%–82% and nodulation upregulated by 51%–61% relative to control. Similarly, leaf photosynthetic capacity was maximized by 23%–32% and gas exchange efficiency by 23%–27% under the n‐MoS_2_ with earthworm treatment. Additionally, NR, NiR, GS, GOGAT, and urease increased 30%–46% in shoots and 23%–43% in roots. Moreover, shoot and root N contents increased by 56%–68%, while nitrogenase activity and BNF increased by 40%–51%, respectively. Consequently, pods, seeds, and grain yield per plant increased by 90, 64% and 41%, respectively. Accordingly, seed protein and soluble sugars improved by 44% and 98%, respectively. Collectively, our findings show that earthworms amplify the efficacy of n‐MoS_2_, mobilizing Mo and strengthening BNF to deliver higher soybean yield and improved seed quality, supporting a practical, low‐input strategy for sustainable production.

## 1 Introduction

Soybean is among the top 10 crops with the highest global production (Zhao et al. 2017; FAO 2024), providing > 28% of vegetable oil and 67% of protein meal, and its production has expanded 13‐fold over the past six decades (Tian et al. 2025). Brazil and the United States lead with 0.8% and 0.5% yearly growth, while Argentina and Paraguay reach 56 and 13 Mt. by 2034, and production in China, India, Russia, Ukraine, and Canada likewise rises (OECD/FAO 2025). Sustaining yield while lowering inputs hinges on nitrogen management. In soybean, a substantial share of plant N derives from biological nitrogen fixation (BNF), which directly influences yield and seed quality while reducing reliance on synthetic fertilizers (Herridge et al. 2008). Therefore, strengthening BNF and improving nitrogen use efficiency (NUE) offers a practical route to maintain productivity and reduce emissions linked to industrial N production (Fowler et al. 2013). Yet, BNF performance is constrained by nodulation homeostasis and redox balance, where excess reactive oxygen species and high O_2_ levels inhibit nitrogenase activity as well as adequate micronutrient supply (Becana et al. 2010; Meilhoc et al. 2025).

Molybdenum (Mo) governs legume N acquisition through Mo‐cofactor enzyme systems that catalyze symbiotic N_2_ reduction and nitrate assimilation (Schwarz and Mendel 2006; Buren et al. 2020). In many agricultural soils, molybdate availability is constrained by pH and redox‐sensitive sorption; as pH approaches neutral under more oxidizing conditions, sorption decreases and plant availability increases (Rutkowska et al. 2017). Together, these control Mo as a key micronutrient linking rhizosphere chemistry to N acquisition in soybean. Translating this need into practical implications highlights the Mo nanomaterial (NM) as a key source that modulates dissolution and residence at the root‐soil interface (Lee et al. 2019).

Nanostructured molybdenum disulfide (n‐MoS_2_) is a 2D Mo source with properties that could stabilize Mo supply to roots. In aqueous, oxygenated environments, n‐MoS_2_ oxidatively dissolves to release molybdate, with kinetics governed by structure and preparation history, suggesting controllable, slow‐release behavior relative to soluble salts (Li et al. 2023). Recent soybean work shows n‐MoS_2_ (10 mg kg^−1^) delays nodule senescence, enhances plant nutrition and BNF, and grain yield by 30% compared with molybdate, indicating that Mo NM can reprogram the symbiosis under realistic conditions (Li et al. 2023). Another study demonstrates that dynamic MoS_2_ transformation within soil–plant systems contributes to bioavailable Mo and S (Li et al. 2024). These findings justify our focus on n‐MoS_2_ and the selected dose. However, it remains unclear how soybean responds to 10 mg kg^−1^ n‐MoS_2_ in the presence of earthworms (EW), which is essential for understanding the rhizosphere mechanisms governing nitrogen fixation and crop performance.

EW are well‐established rhizosphere engineers that restructure pore networks, mix residues, and transport solutes vertically, with consistent positive effects on crop performance (van Groenigen et al. 2014; Fonte et al. 2023). They create mucus and cast‐derived micro zones that shift nitrate availability, moderate acidity, and reshape microbial communities (Blouin et al. 2013). Beyond altering chemistry, earthworm bioturbation physically redistributes NMs through the soil profile, as shown for Ag_2_S NMs in X‐ray CT column experiments (Baccaro et al. 2019). Consistent with this biotic‐amplifier view, silica NMs combined with EW increased soil Si bioavailability and reprogrammed maize root metabolite profiles, indicating that earthworm activity can potentiate nano‐derived nutrient supply and metabolic outcomes (Ma et al. 2021). Across agroecosystems, EW increase crop yield by 25% on average (van Groenigen et al. 2014), contributing to 6.5% of global grain production and 2.3% of legume production, underscoring their relevance for yield formation (Fonte et al. 2023). Such changes could accelerate n‐MoS_2_ oxidation, increase molybdate mobility and plant Mo uptake in legumes. Long‐term field evidence further shows that EW increase ecosystem multifunctionality by strengthening microbial‐microfaunal associations and shifting communities toward a bacterial energy channel (Liu et al. 2019), underscoring their capacity to coordinate rhizosphere biota relevant to nutrient cycling. However, whether EW reshape porewater chemistry to change the speciation and plant availability of Mo from n‐MoS_2_ remains untested.

Despite rapid growth in nano‐enabled agronomy, most studies treat NMs or soil fauna in isolation. We lack an integrated test of how EW modulate n‐MoS_2_ behavior to reconfigure rhizosphere chemistry, redistribute Mo pools across soil‐root‐shoot compartments, and propagate effects through root system architecture, nodulation, leaf gas exchange, nitrogen assimilation enzymes, and yield in a legume system. Addressing this gap is essential for evaluating nano‐inputs, where delivery, transformation, and biological context govern efficacy. Here, we varied n‐MoS_2_ supply (0 or 10 mg kg^−1^) and earthworm presence to isolate NM, earthworm, and interactive effects in soybean. We hypothesized that EW would enrich rhizosphere nitrate and buffer pH toward neutral, accelerating n‐MoS_2_ oxidation and Mo mobilization, thereby increasing Mo translocation to shoots, expanding root system architecture and nodulation, upregulating NR, NiR, GS‐GOGAT, and urease (UE), enhancing nitrogenase activity, and ultimately delivering a number‐driven yield increase. These findings provide mechanistic insight into earthworm and NM‐driven enhancement of crop nutrient utilization.

## 2 Material and Methods

### 2.1 NM Characterization

**FIGURE 1:**
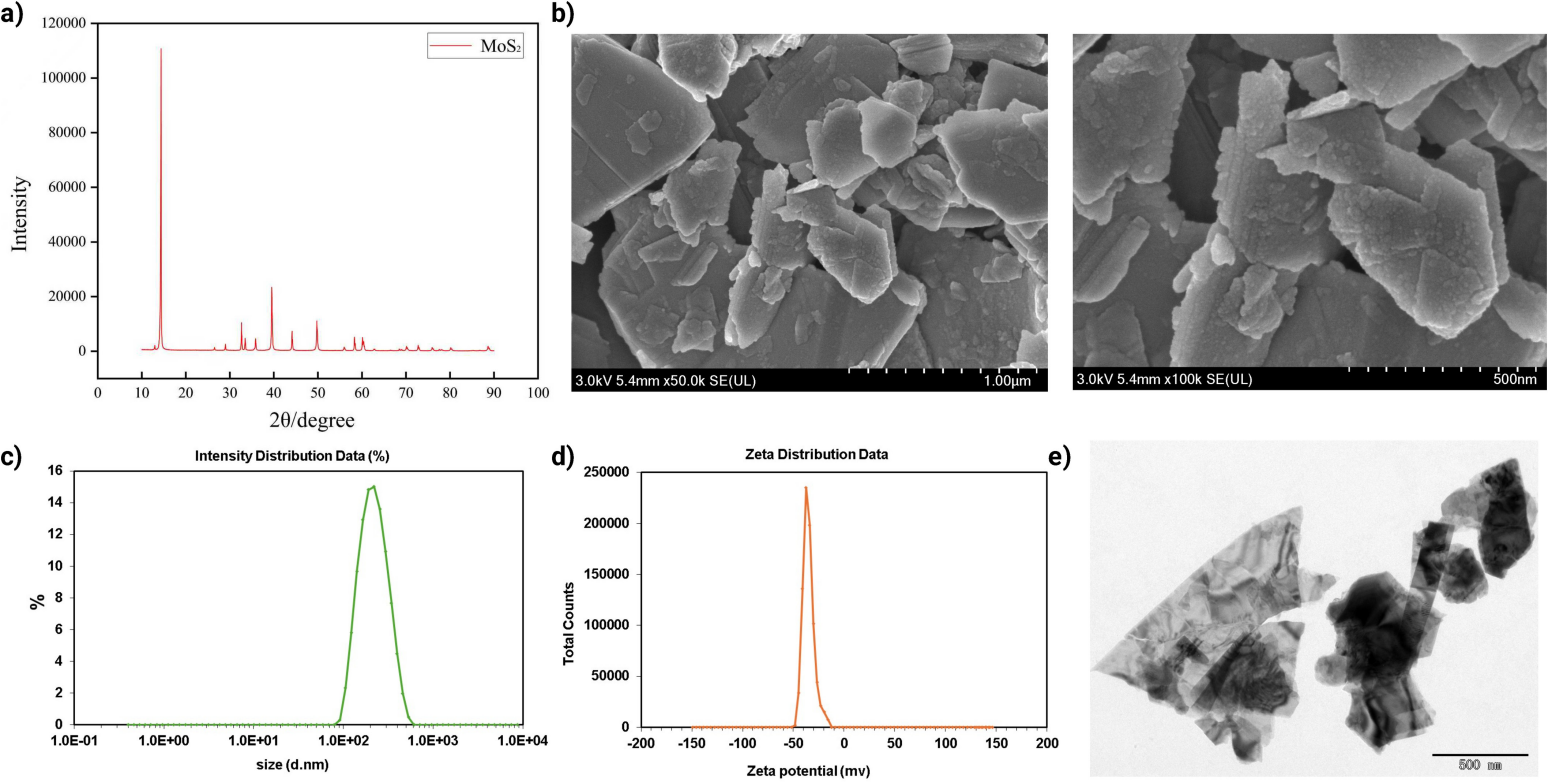
Physicochemical characterization of n‐MoS_2_. (a) Powder X‐ray diffraction pattern indexed to 2H‐MoS_2_ (dominant (002) at ~14° 2θ). (b) FE‐SEM images showing platelet‐like nanosheets (scale bars: 1 μm, 500 nm). (c) Intensity‐weighted hydrodynamic size distribution (DLS). (d) Zeta‐potential distribution. (e) TEM of thin, wrinkled nanosheets (scale bar: 500 nm).

Nano‐Molybdenum (n‐MoS_2_, 99.99% purity) with a supplier‐stated primary size of 80–100 nm was obtained from Guangdong Nanuo Materials Technology Co. Ltd. The morphology features and particle size of n‐MoS_2_ were analyzed using transmission electron microscopy (TEM; JEM‐1200EX, JEOL) and scanning electron microscopy (SEM) (Figure 1). Surface charge (ζ‐potential) and intensity‐weighted Z‐average hydrodynamic diameter were determined via dynamic light scattering (DLS) and electrophoretic light scattering using a Zetasizer Nano ZS90 (Malvern Instruments, UK) equipped with a He‐Ne laser (*λ* = 633 nm) at 25°C (Figure 1). All measurements were performed in triplicate using ultrapure water (pH ~7.0) as the dispersion medium. Powder X‐ray diffraction (XRD; Bruker D8 Advance) was used to assess the crystallographic phase.

### 2.2 Experimental Setup

A two‐factor, complete randomized design (CRD) (n‐MoS_2_ × earthworm treatment) was conducted to investigate the effect of n‐MoS_2_ and earthworm inclusion on soybean growth and nitrogen assimilation. The n‐MoS_2_ treatment comprised 0 mg kg^−1^ (Ctrl) or 10 mg kg^−1^ n‐MoS_2_; with and without earthworm (EW), with this concentration previously established as agronomically relevant and non‐phytotoxic in soybean (Li et al. 2023). Each treatment had eight replicates. To enable stage‐wise comparisons, four replicate pots per treatment were destructively harvested at the vegetative stage, and the remaining four were grown to full maturity to obtain seeds. The study was carried out using soil collected from the Shangzhuang Experimental Station of China Agricultural University (Beijing, China; 40°08′12′ N, 116°10′48′ E). The soil, classified as silt loam, was air dried, homogenized, and sieved through a 2 mm mesh to standardized particle size. The physicochemical properties of the processed soil were analyzed, and detailed information is provided in Table S1. Soybean ( *Glycine max* ) seeds (zhonghuang 13) were obtained from Fengshi Seed Industry Company. Seeds were sterilized by immersion in 3% sodium hypochlorite solution for 10 min, followed by 3 rinses with deionized water. After sterilization, seedlings were pre‐germinated. The seedlings were then transferred into plastic pots with 2 kg soil per pot, following a CRD. Earthworm ( *Eisenia fetida* ; mean individual mass 0.60 ± 0.05 g fresh weight) were purchased from Yilong Earthworm Farm Co. Ltd. Prior to the experiment, EW were acclimated in uncontaminated soil for 7 days at 20°C ± 2°C under a 16:8 h light/dark cycle. For the earthworm treatment, 20 individuals were added per pot. Earthworm recovery at harvest was 90% in the EW treatment and 80% in the n‐MoS_2_ + EW treatments (Table S2). The experiment was conducted in a climate‐controlled greenhouse at 25°C ± 1°C (day/light), with a relative humidity of 70%. Soil moisture was maintained at 60% field capacity using distilled water throughout the experiment. Plants were harvested at two predefined times: 60 days after planting (DAP) for nodulation and nitrogen‐process assays and 120 DAP (physiological maturity) for yield and seed quality. Porewater was sampled non‐destructively at 30, 60, and 90 DAP using suction samplers connected to luer‐lock syringes, immediately cooled (4°C), and analyzed for pH, electrical conductivity (EC), NO_3_^−^‐N, and NH_4_^+^‐N.

### 2.3 Harvesting and Measurement of Plant and Root Parameters

Plants were carefully harvested to assess various phenotypic and physiological parameters. The following plant growth traits were measured: plant height, shoot dry biomass (60°C to constant mass), number of pods, number of seeds, seed weight, and 100‐seed weight. For root parameters, the plant was carefully uprooted, and roots were washed thoroughly to remove soil. The following root parameters were determined: root length, root mass, average root diameter, total root volume, total surface area, number of nodules, and nodule weight.

### 2.4 Mo Analysis in Soil and Soybean Plant Tissue

Mo concentration in soil and soybean plant tissue (shoot and root) was determined by microwave digestion followed by analysis using inductively coupled plasma mass spectrometry (ICP‐MS; EXPEC 7200, EXPEC Technology) (Li et al. 2023). The translocation factor for Mo content was calculated as TF = [Mo]shoot/[Mo]root. Additionally, the Mo concentrations in rhizosphere (root‐adhering) soil were analyzed after aqua regia digestion using a microwave digestion system (Azeem et al. 2024).

### 2.5 Photosynthetic Efficiency and Chlorophyll Content Measurement

Photosynthetic efficiency, including the net photosynthesis rate (Pn), stomatal conductance (Gs), intercellular carbon dioxide concentration (Ci), and transpiration rate (Tr), was measured using an open gas‐exchange system (LI‐COR Biosciences) on the most recently fully expanded leaf between 09:00 and 11:30, at PPFD 1000 μmol m^−2^ s^−1^ and reference CO_2_ 400 μmol mol^−1^. The relative chlorophyll content (SPAD index) was measured at 10 points near the main vein of the same leaf to assess the chlorophyll content. Gas‐exchange and SPAD were measured on the cohort designated for the corresponding harvest within 1 day prior to harvest (Azeem et al. 2024).

### 2.6 Nitrogen Assimilation and Nitrogenase Activity Assays

The enzymatic activity of nitrite reductase (NiR), nitrate reductase (NR) assay kit (A096‐1‐2), glutamate synthetase (GOGAT) assay kit (H625‐1‐2), and glutamine synthetase (GS) assay kit (A047‐1‐2) in root and shoot was measured according to the manufacturer's protocols (Nanjing Jiancheng Bioengineering Co. Ltd.). UE activity in the root and shoot was determined using assay kits (Beijing Boxbio Co. Ltd. AKNM003M) (Zhou et al. 2023). Nitrogen content in soybean root and shoot (dried powder) was analyzed using an elemental analyzer (Elementar Vario EL) (Shakoor et al. 2023). Nitrogenase activity in the nodule was quantified using an acetylene reduction assay. Fresh nodules were placed in 50 mL serum vials; headspace was adjusted to 10% (v/v) acetylene. The vials were incubated for 30 min at 30°C; after that, 500 μL gas was sampled and analyzed for ethylene content using gas chromatography (Agilent 7890). Nitrogenase activity was reported as μmol C_2_H_4_ g^−1^ FW h^−1^.

### 2.7 Organic Nutrient Analysis

Soluble protein content was measured using a total protein assay kit (A045‐3‐2) (Nanjing Jiancheng Co.), following the manufacturer's protocol. Soluble sugar and starch contents were determined by the anthrone colorimetric method (Li et al. 2023).

### 2.8 Data Analysis

The data are presented as mean ± standard deviation (SD) of four replicates per treatment per stage (*n* = 4 per treatment × stage; total *n* = 8 per treatment across stages). Vegetative and maturity datasets were analyzed separately. Within each stage, two‐way ANOVA (factors: n‐MoS_2_ and EW) with Tukey's HSD (*α* = 0.05) was used. Analyses were performed using Statistix 8.1 (Analytical Software). GraphPad Prism 10 was used to construct the figures.

## 3 Results

### 3.1 Physicochemical Characterization of n‐MoS_2_

Powder X‐ray diffraction confirmed phase‐pure 2H‐MoS_2_ with a dominant (002) reflection at 14° 2θ (*d* ≈ 0.62 nm) and higher‐order basal/prismatic reflections (Figure 1a), notably indicating layered stacking typical of few to multi‐layer flakes without detectable oxide impurities. Consistently, FE‐SEM resolved plate‐like, lamellar particles with stepped edges and sub‐micrometer to few‐micrometer lateral dimensions (Figure 1b), and TEM corroborated thin, wrinkled nanosheets with overlapping platelets (Figure 1e), thereby confirming a 2D flake architecture rather than spherical particulates. Complementarily, DLS of aqueous dispersions showed a unimodal intensity peak in the 10^2^‐nm range (Figure 1c), indicating sub‐micrometer hydrodynamic entities under the measurement conditions. However, because intensity weighting overemphasizes larger objects and platelets diffuse anisotropically, we interpret DLS as a comparative dispersion descriptor that supplements lateral size estimates from microscopy. Moreover, the ζ‐potential centered near −40 mV (Figure 1d), which is consistent with electrostatic stabilization in low‐ionic‐strength media. Collectively, the crystallographic, microscopic, and colloidal data establish n‐MoS_2_ as well‐crystallized, electrostatically stabilized 2D flakes, thereby providing a consistent physicochemical baseline for subsequent biological assays.

### 3.2 Mo‐NMs and EW Reconfigure Rhizosphere Chemistry

**FIGURE 2:**
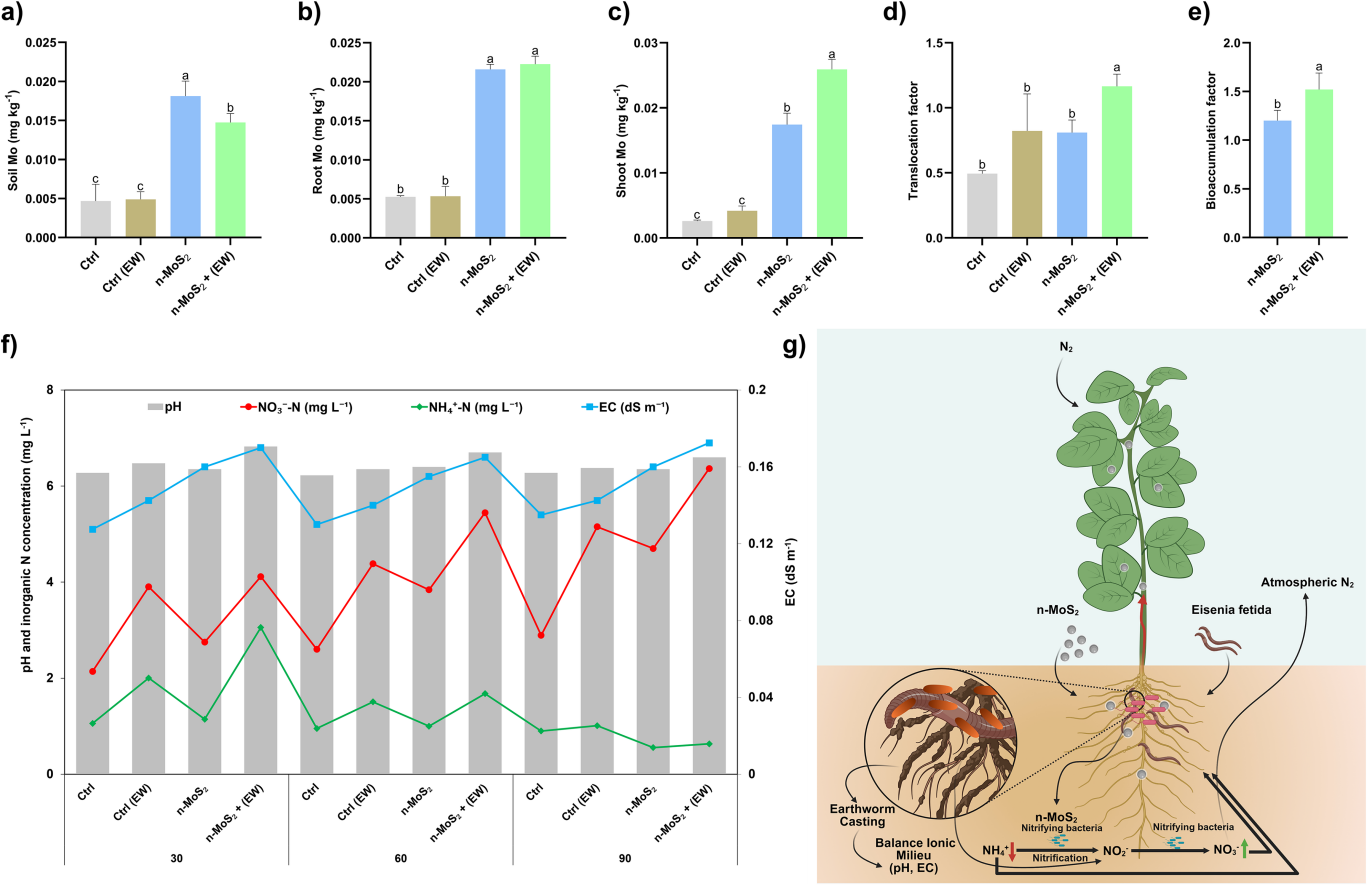
Mo concentrations across soil, root, and shoot, transport indices, and rhizosphere chemistry under n‐MoS_2_ with and without earthworms (a) Soil Mo, (b) root Mo, and (c) Shoot Mo. (d) Translocation factor. (e) Bioaccumulation factor. Bars show mean ± SD (*n* = 4); different letters indicate significant differences among treatments (Two‐way ANOVA, Tukey, *p* < 0.05). Treatments: Ctrl (control), Ctrl (EW), n‐MoS_2_, and n‐MoS_2_ + (EW). (f) Rhizosphere pH, inorganic nitrogen concentration (NO_3_^−^‐N and NH_4_^+^‐N; mg L^−1^), and electrical conductivity (EC; dS m^−1^) at 30, 60, and 90 DAS. Gray bars represent pH, red circles represent NO_3_^−^‐N, green diamonds represent NH_4_^+^‐N, and blue squares represent EC. (g) Schematic working model: Earthworms alter pH/EC and NO_3_^−^/ NH_4_^+^, consistent with Mo release from n‐MoS_2_ and enhanced root‐shoot transfer.

To frame subsequent biological details, we characterized Mo concentration and accompanying rhizosphere conditions under n‐MoS_2_ with and without EW. To quantify the distribution of Mo supplied as n‐MoS_2_ within the soil–plant system, we measured Mo in soil, roots, and shoots, computed translocation and bioaccumulation indices, and tracked rhizosphere pH, NO_3_^−^‐N, NH_4_^+^‐N, and EC over time (Figure 2). Under co‐application of n‐MoS_2_ and EW, shoot Mo increased by 49%, soil Mo decreased by 19%, and root Mo was essentially unchanged (3% lower) relative to n‐MoS_2_ alone (Figure 2a–c). Furthermore, co‐application increased the translocation and bioaccumulation factors by 44% and 26%, respectively, relative to n‐MoS_2_ (Figure 2d,e). Rhizosphere chemistry (pH, NO_3_^−^‐N, NH_4_^+^‐N, EC) was monitored at 30, 60, and 90 days to further contextualize the Mo pools (Figure 2f). Across time, pH showed slight shifts toward neutral under co‐application, remaining within 6.2–6.8. NO_3_^−^‐N increased over the course of the experiment and was consistently highest with co‐application. Values elevated from 4.11 mg L^−1^ at 30 d to 5.44 mg L^−1^ at 60 days and 6.36 mg L^−1^ at 90 days, exceeding the corresponding n‐MoS_2_ values at each timepoint. In contrast, NH_4_^+^‐N declined under co‐application from 3.06 to 0.63 mg L^−1^ between 30 and 90 days. Electrical conductivity remained within a narrow range but was slightly higher with co‐application (0.170, 0.165, 0.173 dS m^−1^ at 30, 60, 90 days, respectively) than with n‐MoS_2_. Consistent with these measurements, a schematic in Figure 2g outlines a plausible pathway in which earthworm‐driven changes to the rhizosphere favor Mo mobilization from n‐MoS_2_ and its subsequent translocation.

### 3.3 Root System Architecture and Symbiotic Nodulation

**FIGURE 3:**
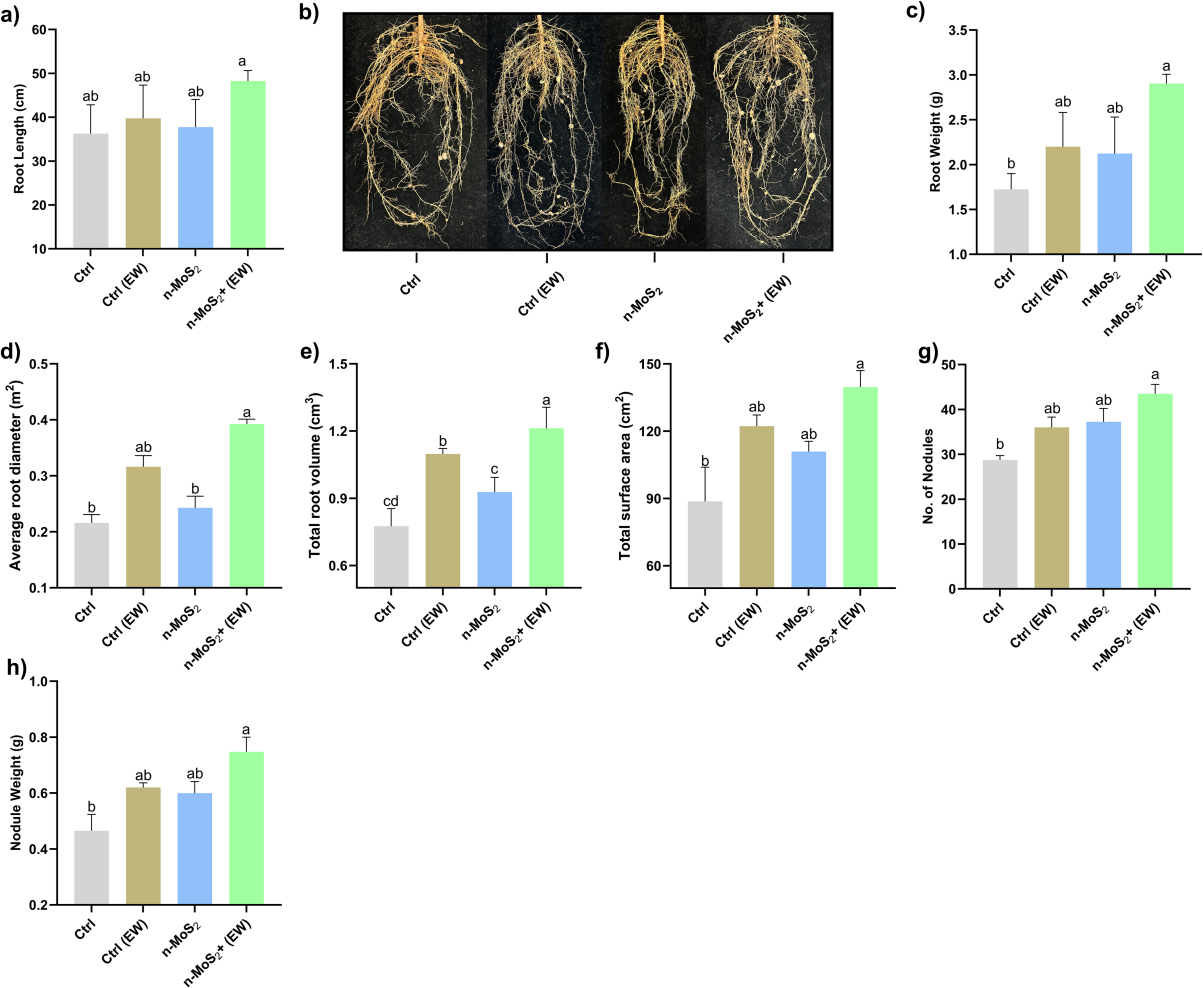
Earthworms enhance root architecture and symbiotic nodulation under n‐MoS_2_ exposure. Root system and nodulation of soybean. (a) Root length, (b) Root morphology, (c) Root fresh weight, (d) average root diameter, (e) Total Root volume, (f) Total root surface area, (g) Number of nodules, and (h) Nodule fresh weight. Bars show means ± SD (*n* = 4). Different letters denote significant differences among treatments (Two‐way ANOVA, Tukey's HSD, *p* < 0.05).

We evaluated soybean roots under four treatments to test whether EW translate the benefit of n‐MoS_2_ into tangible belowground responses. Representative root images show a clear shift from sparse, fine roots in Ctrl to thicker axes, greater lateral branching, and visibly more nodules under n‐MoS_2_ + EW (Figure 3b). Notably, EW‐assisted n‐MoS_2_ delivered the greatest and statistically supported improvements across all metrics (*p* < 0.05), root length elevated by 33%, root mass by 63%, total surface area by 57%, total volume by 56%, and average diameter by 82% relative to Ctrl (Figure 3a–f). Nodulation increased concordantly, with nodule number and nodule fresh weight 51% and 61% higher than Ctrl, respectively (Figure 3g,h). Consequently, these effects support a larger absorptive interface and higher symbiotic capacity, conditions compatible with enhanced BNF. Additionally, EW alone strengthened multiple root architectural features versus Ctrl, including root length (10%), mass (24%), surface area (38%), volume (42%), and diameter (47%). Similarly, nodulation also increased, with nodule number up by 25% and nodule mass by 33%. Moreover, n‐MoS_2_ alone produced moderate boosts of root mass (20%), surface area (25%), volume (20%), diameter (13%), nodule number (30%), and nodule mass (29%), while its effect on root length remained non‐significant (4%). In contrast, neither single input matched the breadth or magnitude of the co‐application. For example, relative to the single‐factor treatments, n‐MoS_2_ + EW yielded equal or greater values for average root diameter, total root volume, and root fresh weight, where letters differ. Nodulation (number and fresh weight) under co‐application was maintained or higher than with EW alone.

### 3.4 Leaf Gas‐Exchange Responses

**FIGURE 4:**
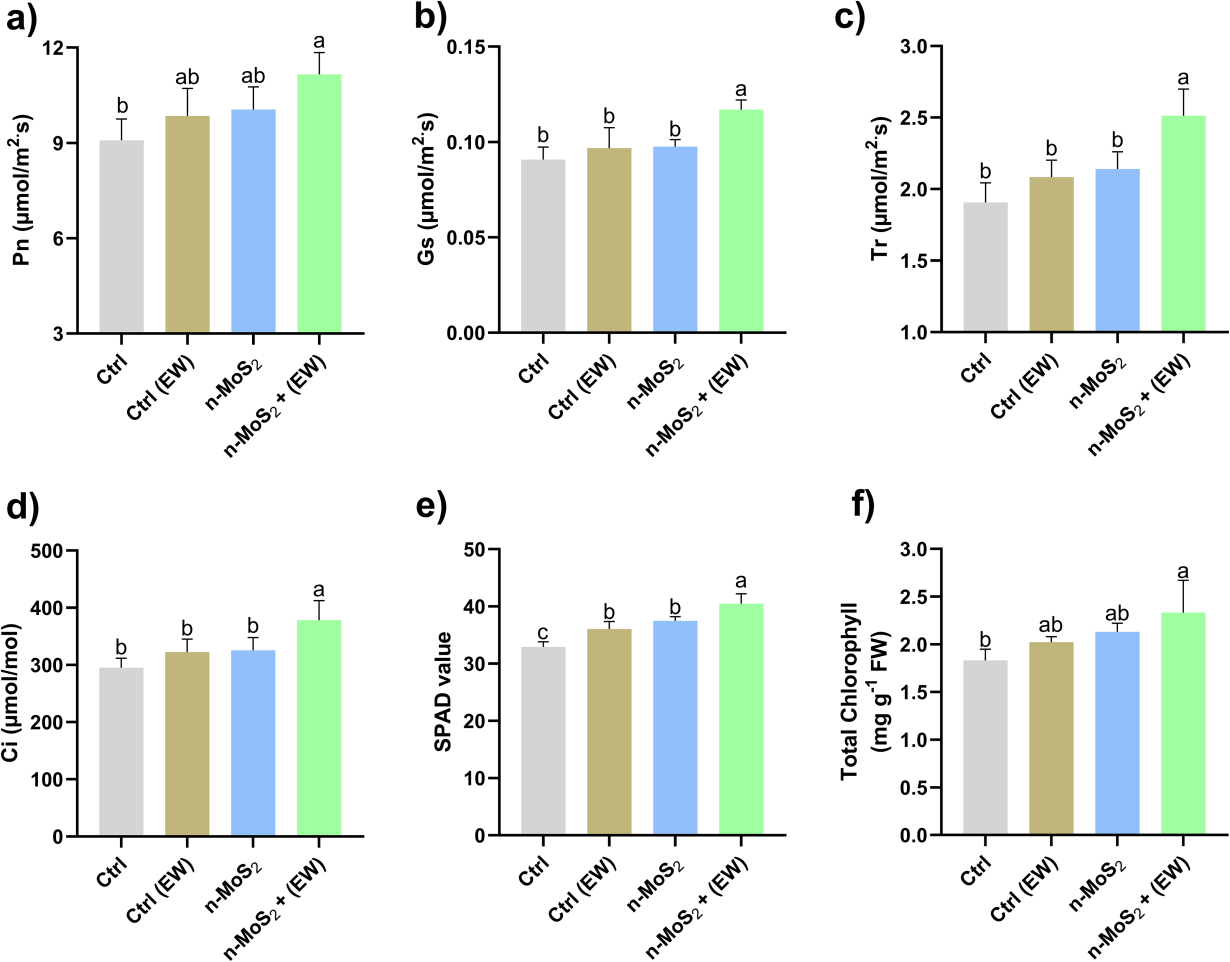
Leaf gas‐exchange responses of soybean. (a) Net photosynthetic rate; (b) Stomatal conductance; (c) Transpiration rate; (d) Intercellular CO_2_ concentration; (e) SPAD chlorophyll index value; (f) Total Chlorophyll. Bars represent means ± SD (*n* = 4); different letters indicate significant differences among treatments (Two‐way ANOVA, Tukey's HSD, *p* < 0.05).

Leaf gas exchange was assessed as net photosynthetic rate (Pn), Gs, transpiration (Tr), intercellular CO_2_ (Ci), and SPAD chlorophyll index. Notably, the photosynthetic traits of soybean exhibited a marked enhancement under all treatments compared with the Ctrl. The Ctrl (EW) treatment slightly improved photosynthetic performance, with moderate increases in Pn, Gs, Ci, and Tr by approximately 7%–10%, reflecting a mild stimulatory effect of the external amendment (Figure 4a–d). Exposure to n‐MoS_2_ further elevated these parameters by about 10%–12%, suggesting improved CO_2_ assimilation and stomatal regulation under NMs influence. SPAD chlorophyll index and total chlorophyll increased by 23% and 27% with n‐MoS_2_ + EW relative to Ctrl (Figure 4e,f), respectively, showing similar patterns with the gas exchange traits. The combined treatment n‐MoS_2_ + EW induced the most pronounced responses, enhancing Pn, Ci, and Tr by nearly 23%–32% relative to the Ctrl (Figure 4a–d), indicating the strongest combined response under n‐MoS_2_ + EW that optimized photosynthetic capacity and gas exchange efficiency in soybean leaves. Because gas‐exchange patterns can reflect nitrogen status, we next quantified nitrogen‐assimilation enzymes.

### 3.5 Nitrogen‐Assimilation Enzyme Activity

**FIGURE 5:**
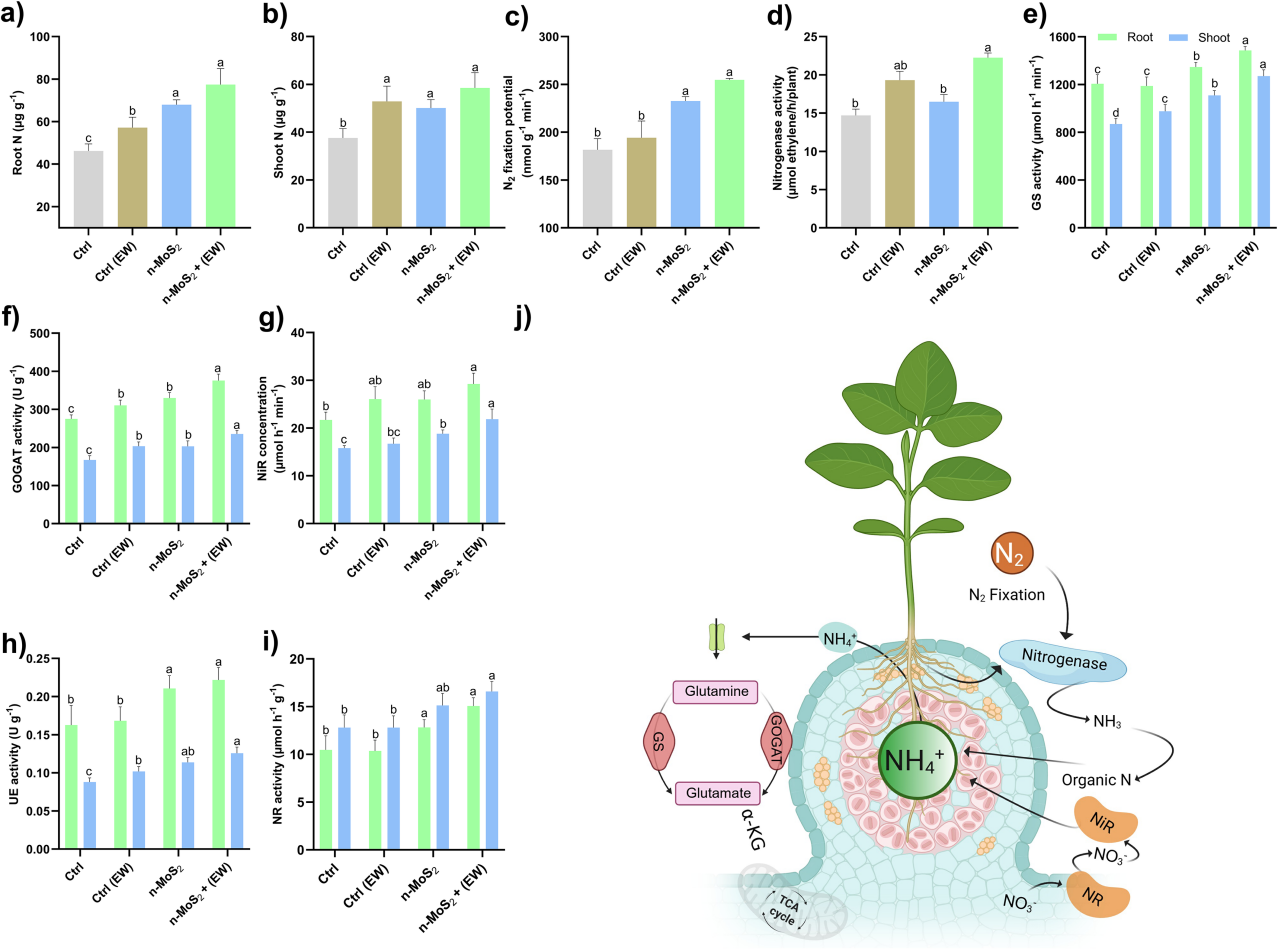
Nitrogen assimilation and enzyme activity in soybean under Ctrl, Ctrl (EW), n‐MoS_2_, and n‐MoS_2_ + (EW) treatments. (a) Root nitrogen content; (b) Shoot nitrogen content; (c) Nitrogen fixation potential; (d) Nitrogenase activity; (e) Glutamine synthetase (GS) activity; (f) Glutamate synthase (GOGAT) activity; (g) Nitrite reductase (NiR) activity; (h) Urease (UE) activity; (i) Nitrate reductase (NR) activity. Bars represent means ± SD (*n* = 4); different letters indicate significant differences among treatments (Two‐way ANOVA, Tukey's HSD, *p* < 0.05). (j) depicts a schematic of the nitrogen fixation process, illustrating the roles of key nitrogen metabolic pathways and enzymes.

To test whether the stronger nodulation translates into functional nitrogen supply and use, we report the results in the order shown in Figure 5: tissue nitrogen, fixation capacity, and the assimilatory enzymes, followed by the pathway schematic. Additionally, nitrogen content in both root and shoot tissues was elevated with co‐application. Root N (Figure 5a) showed an approximate increase of 68% relative to the Ctrl, while shoot N (Figure 5b) significantly increased by 56%. These increases were consistent with the upregulation of nitrogen‐assimilation enzymes. Moreover, nitrogen fixation potential (Figure 5c) showed a marked increase under co‐application, with values elevated by 40% compared to Ctrl, indicating a strengthened nitrogen fixation process in soybean under the combined influence of EW and n‐MoS_2_. Consistent with this, nitrogenase activity (Figure 5d) was significantly enhanced by co‐application, with values reaching 51% higher than the Ctrl. Co‐application of EW and n‐MoS_2_ remarkably enhanced nitrogen assimilation in soybean, as evidenced by the coordinated upregulation of nitrogen‐related enzymes in both shoots and roots. Compared with the Ctrl, shoot GS, GOGAT, NiR, UE, and NR activities (Figure 5e–i) increased by approximately 30%–46%, highlighting a strong stimulation of the enzymatic mechanism governing nitrogen metabolism. Similarly, root enzymatic activities followed a comparable pattern, with GS, GOGAT, NiR, UE, and NR (Figure 5e–i) showing significant enhancements of about 23%–43%, suggesting improved nitrogen reduction and assimilation capacity under the synergistic influence of NMs and biological activity. Moreover, the n‐MoS_2_ treatment alone induced a moderate upregulation of these enzymes (12%–29%) in both shoot and root tissues, indicating the potential of Mo‐based NMs to enhance nitrogen turnover independently. In contrast, the EW treatment exhibited only slight improvements (5%–10%) in most enzymes, indicating smaller changes relative to the co‐application. Enzyme activity ranking mirrored the nodulation patterns, indicating that co‐application enhances nitrogen assimilation and BNF. The underlying mechanisms, including N_2_ fixation and nitrogen assimilation, are illustrated in Figure 5j.

### 3.6 Growth, Yield Formation, and Allocation

**FIGURE 6:**
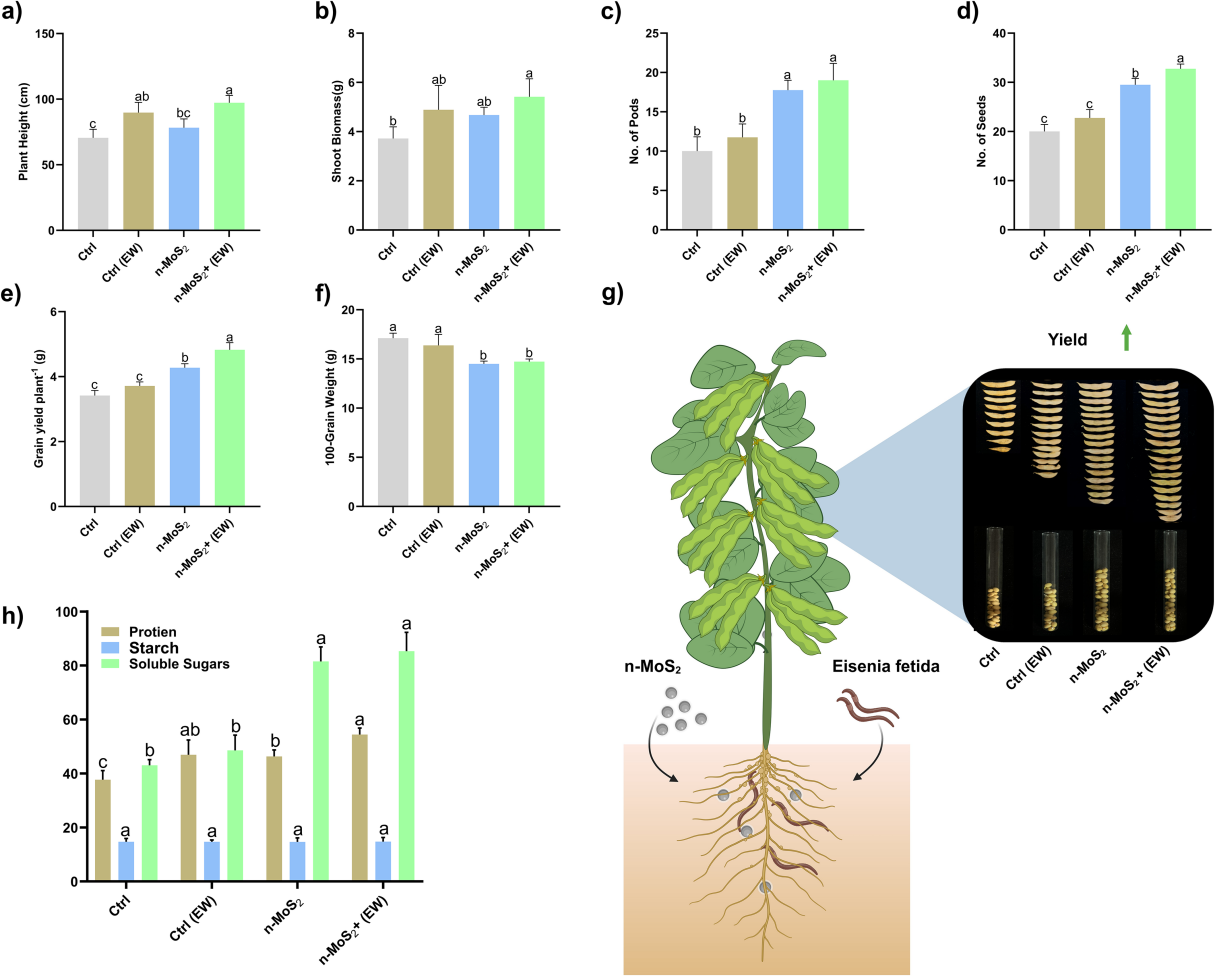
Growth, reproductive performance, and biochemical content in soybean under Ctrl, Ctrl (EW), n‐MoS_2_, and n‐MoS_2_ + (EW). (a) Plant height; (b) Shoot biomass; (c) Number of pods; (d) Number of seeds; (e) Grain weight; (f) 100‐grain weight; (g) Yield images showing the harvest outcome for each treatment (biorender.com); (h) Protein, starch, and soluble sugars contents. Bars represent means ± SD (*n* = 4); different letters indicate significant differences among treatments (Two‐way ANOVA, Tukey's HSD, *p* < 0.05). Panel (g) was created using BioRender.com.

We assessed the whole plant performance to determine whether EW translates the n‐MoS_2_ signal into yield improvements. Notably, the co‐application outperformed all other treatments across performance metrics, augmenting plant height by 38% and shoot biomass by 46% (Figure 6a,b). Moreover, reproductive capacity expanded sharply, with pods and seeds per plant increasing by 90% and 64% (Figure 6c,d), respectively; these gains were larger than those from either input alone. Furthermore, grain weight per plant rose by 41%, indicating that the enlarged reproductive sink converted into measurable harvest (Figure 6e). In contrast, 100‐grain weight declined by 14% under the co‐application (Figure 6f), mirroring the reduction under n‐MoS_2_ alone and the modest decrease with EW, a classic seed number‐size trade‐off. Additionally, single‐factor treatments produced moderate improvements: EW increased height and biomass by 27% and 32%, while n‐MoS_2_ raised biomass by 26% and strongly boosted pods and seeds by 78% and 48%. The co‐application exceeded these responses in magnitude or breadth, particularly for reproductive counts. Protein and soluble sugars (Figure 6h) also showed significant increases under co‐application, with protein improving by 44%, soluble sugars by 98% relative to Ctrl, while starch remained unchanged. Consequently, the data reveal a coordinated enhancement of vegetative growth and reproductive allocation when EW and n‐MoS_2_ are combined, with yield gains driven primarily by more pods and more seeds rather than heavier seeds (Figure 6g). Therefore, integrating soil fauna with a nano‐enabled amendment offers a practical route to upscale harvest outcomes in soybean. Together, these components indicate a number‐driven yield increase under n‐MoS_2_ + EW.

## 4 Discussion

The integration of EW and n‐MoS_2_ has proven to be a prevailing strategy for improving soybean growth, nitrogen fixation, and yield formation. Our results demonstrate that the co‐application of EW and n‐MoS_2_ led to significant improvements in rhizospheric chemistry, photosynthetic capacity, root health, nodulation, and overall yield. These enhancements were primarily driven by increased Mo availability, improved nitrogen metabolism, and better soil structure, resulting in a synergistic effect that surpassed the benefits of either treatment alone. In this section, we explore the mechanisms underlying these improvements and their implications for sustainable crop production. To our knowledge, this is the first explicit demonstration that earthworm activity modulates n‐MoS_2_ in soil to increase plant‐available Mo, strengthen BNF, and raise yield at an agronomic dose.

### 4.1 EW Enhance Mo Release and Shoot Uptake From n‐MoS_2_

The co‐application produced small shifts toward neutral pH and slightly higher EC, together with a progressive rise in NO_3_^−^‐N and decline in NH_4_^+^‐N. Such chemistry is consistent with two mutually reinforcing earthworm functions. First, EW are ecosystem bio‐engineers whose calciferous glands secrete CaCO_3_ granules and whose casts and mucus buffer microsite pH; both effects can favor molybdate (MoO_4_^2−^) mobility because Mo sorption onto Fe/Al oxides declines as pH approaches neutrality (Singh et al. 2016). Second, earthworm activity commonly accelerates nitrification by stimulating ammonia‐oxidizing archaea/bacteria (AOA/AOB), explaining the NO_3_^−^‐N rise and NH_4_^+^‐N drawdown seen here (Huang et al. 2017). Therefore, the net effect is a rhizosphere with slightly higher ionic strength and oxygenation, more nitrate supply, and more available Mo, a similar mechanism reported earlier in which slow‐release n‐MoS_2_ can be converted into physiologically useful Mo and S over the time window that plants need it (Kaiser et al. 2005; Li et al. 2024), with translocation and bioaccumulation factor indicating earthworm‐driven routing of Mo from soil reservoirs to shoots rather than passive accumulation.

Mechanistically, these soil shifts align with our pool measurements, such as relative to n‐MoS_2_ alone. Adding EW decreased soil Mo (19%) yet increased shoot Mo (49%) without materially changing root Mo and raised both the translocation and bioaccumulation indices (by 44% and 26%). We interpret this as earthworm‐mediated enhancement of (i) microsite transformation/oxidation of n‐MoS_2_ and (ii) root‐particle‐microbe encounter frequency within burrow walls and casts, improving delivery of Mo to the vascular stream. Together, these processes provide the chemical and physical framework for the biological responses observed below (nodulation, N‐enzyme activation, photosynthesis, and yield) with casts and burrow walls acting as microsites that accelerate n‐MoS_2_ transformation and increase root‐particle‐microbe encounters.

### 4.2 EW Elevate n‐MoS_2_ Effects on Roots, Nodules, and N Assimilation

The coordinated improvements in root length, biomass, surface area, and volume under co‐application (vs. either single factor) indicate that EW converted the nano‐signal into a larger absorptive interface. EW bioturbation and casting generate nutrient‐rich, well‐aerated microsites, while their movement enhances rhizobia dispersal and the probability of successful infection events; a previous study has shown that EW can facilitate the spread of *Rhizobium/Bradyrhizobium* and increase nodulation (Li et al. 2021). Within this physical framework, the Mo released from n‐MoS_2_ addresses the primary biochemical linkage in N metabolism; for instance, NR and nitrogenase both require Mo cofactors; GS/GOGAT then incorporate reduced *N* into amino acids (Li et al. 2023). Consistent with that framework, co‐application significantly elevated NR, NiR, GS, GOGAT, and UE activities in both roots and shoots (23%–46%) and boosted nitrogenase activity and fixation potential (40%–51%), mirroring the higher nodule number and mass, effectively converting n‐MoS_2_ into a fauna‐amplified nanofertilizer at 10 mg kg^−1^. These findings align with recent soybean studies showing that MoS_2_ nanofertilizer at low dose (10 mg kg^−1^) delays nodule senescence, enhances biological N_2_ fixation, and increases grain yield relative to soluble molybdate (Li et al. 2023). They also align with evidence that n‐MoS_2_ can both release small amounts of Mo and S into enzyme pools and persist as an antioxidant “nanozyme” fraction that buffers redox stress at the root‐nodule interface, conditions favorable for maintaining leghemoglobin function and nitrogenase in a micro‐oxic state over a longer period (Chen et al. 2018), providing a mechanistic basis for delayed nodule senescence and sustained BNF in our system. The outcome is a virtuous cycle where improved root architecture expands the capture zone for nitrate and rhizobia, Mo availability lifts NR/nitrogenase bottlenecks and ROS control helps nodules remain physiologically more active.

### 4.3 EW Enable n‐MoS_2_ to Sustain Photosynthesis and Increase Yield

Leaf‐level responses show the classical signature of N‐driven photosynthetic enhancement; for instance, Pn, Gs, and Tr all increased, Ci remained stable or slightly lower, and SPAD chlorophyll index increased, consistent with a stronger biochemical sink (Rubisco/electron transport) rather than a purely stomatal effect. In C_3_ leaves, N investment is dominated by thylakoid proteins and Rubisco; improving *N* status raises both Rubisco content/activation and electron transport capacity, thereby increasing *V*_cmax_ and *J*_max_ and mesophyll conductance (Luo et al. 2021). The SPAD increase provides a compatible, nondestructive proxy for higher chlorophyll/N status when interpreted with appropriate calibration (Brown et al. 2022). At the whole‐plant level, these sustained source gains translated into higher pod and seed numbers (90% and 64%) and greater grain per plant (41%), with a modest decrease in 100‐seed weight, an expected compensation when sink number expands faster than per‐seed filling capacity over a fixed season (Monzon et al. 2021). Yield increase was associated with coordinated enhancement in nitrogen fixation, photosynthetic capacity, and nutrient uptake, which together supported substantial pod and seed production. This study provides evidence linking the soil‐root‐yield chain for earthworm‐assisted n‐MoS_2_, linking rhizosphere modulation to number‐led yield formation without additional synthetic N. This number‐led yield formation is precisely the outcome anticipated when (i) Mo limitation to NR and nitrogenase is removed and (ii) canopy photosynthesis is protected from ROS‐mediated down‐regulation during the critical seed‐set and fill windows (Li et al. 2023; Li et al. 2024). From an agronomic perspective, this synergy suggests that earthworm‐promoting practices (e.g., reduced tillage, residue retention), which enhance soil structure and microsite chemistry, can be paired with a low‐rate, slow‐release Mo nanofertilizer to achieve yield gains without additional synthetic nitrogen input. This approach is consistent with evidence that EW contribute substantially to crop production (Fonte et al. 2023), supporting a biota‐compatible, low‐dose n‐MoS_2_ approach for sustainable practices. It should be noted that the relatively small pot size (2 kg soil) used in this study may have constrained root system elongation during the 120‐day experiment. Because root development underpins nodulation, nutrient uptake, and yield formation, this physical limitation may have influenced the magnitude of treatment effects relative to field conditions. Therefore, these results should be interpreted with caution, and validation under field conditions is required. In addition, because BNF is tightly regulated by rhizosphere microbial communities, the lack of direct assessment of microbial composition and functions represents a limitation of this study. Future studies integrating microbial profiling with plant physiological response will be necessary to elucidate the mechanisms driving enhanced nitrogen fixation.

## 5 Conclusion

The collective evidence supports a working model in which EW restructure the rhizosphere (micro‐pH/ionic moderation, continuous mineralization without NO_3_^−^ accumulation, inoculum redistribution), while n‐MoS_2_ provides a gradually accessible Mo source via partial oxidative weathering to MoO_4_^2−^, together, these influences promote early infection, sustain nodule function, and elevate N assimilation, which in turn supports higher leaf photosynthetic capacity and ultimately translates into number‐driven yield gains. The generality of fauna‐enabled nano‐nutrient delivery is reinforced by crop studies beyond Mo. For instance, EW have been shown to restructure soil microbiomes under NMs exposure and to amplify the plant responses to nano‐inputs such as silica (Ma et al. 2021), providing a conceptual bridge between our findings and broader soil‐fauna‐NM interactions. Critically, this model is consistent with the nitrate‐inhibition reports where high inorganic N diminishes BNF via Fe handling and nitrate‐signaling pathways and with the Mo fate that documents MoS_2_ oxidation to molybdate in environmental media and soil–plant systems (Zhou et al. 2024).

The interpretations presented here are constrained by the specific condition tested: an n‐MoS_2_ dose of 10 mg kg^−1^, the soil type and pH used, the EW species and density applied, and the background Mo status of the soil. Within this window, the pattern of responses is internally consistent with a BNF‐centered mechanism. Under the tested conditions, earthworm‐assisted n‐MoS_2_ appears compatible with soil biota and dose‐efficient as a strategy to support nitrogen economy in legumes. The co‐application‐maintained rhizosphere conditions that are conducive to nodulation by limiting nitrate‐mediated suppression, delivering bioavailable Mo at a low application rate, and expanding the infection‐competent root surface area. These changes collectively enhanced nitrogen assimilation capacity and canopy photosynthesis, ultimately translating into greater pod and seed set. From an agronomic management perspective, these findings support the use of soil fauna in combination with nano‐enabled micronutrient inputs as a viable approach to improving legume productivity at low dose rates. Future study systematically varying soil type, n‐MoS_2_ dose and earthworm density will refine the operational window for field‐scale deployment, while explicit Mo speciation and NM‐organism interaction studies will strengthen the mechanistic basis of these observations.

## Author Contributions

**Muhammad Nadeem:** conceptualization, methodology, software, formal analysis, investigation, data curation, visualization, writing – original draft, Writing – review and editing, validation. **Muhammad Adeel:** conceptualization, writing – review and editing. **Usama Zaheer:** methodology, writing – original draft, writing – review and editing. **Noman Shakoor:** methodology, writing – original draft, writing – review and editing. **Imran Azeem:** methodology, formal analysis. **Taiming Zhang:** methodology, formal analysis. **Yukui Rui:** conceptualization, investigation, resources, supervision, funding acquisition, writing – review and editing.

## Funding

This work was supported by National Key Research and Development Program of China, 2017YFD0801300, 2017YFD0801103.

## Conflicts of Interest

The authors declare no conflicts of interest.

## Supporting information


**Table S1:** Soil physiochemical properties.
**Table S2:** Earthworm recovery at harvest.

## Data Availability

The data that support the findings of this study are available on request from the corresponding author. The data are not publicly available due to privacy or ethical restrictions.
