# Supplementary material for: Earthworms With Low Dose n‐MoS2 Improve Soybean Yield and Quality by Reconfiguring Rhizosphere Chemistry and Strengthening Nitrogen Fixation
Source: Physiol Plant. 2026 Jun 28;178(4):e70988. doi: 10.1111/ppl.70988 (PMC13310586; doi:10.1111/ppl.70988)
Supplement: Supplementary file 1 — Table S1: Soil physiochemical properties. Table S2: Earthworm recovery at harvest. [file PPL-178-e70988-s001.docx]

**Supplementary Data**

**Table S1:** Soil physiochemical properties.

| Index | Unit | Mean Value |
| --- | --- | --- |
| Soil Texture | - | Silty loam |
| Soil Density | g cm^-3^ | 1.29 |
| pH |  | 6.9 |
| Electrical Conductivity | (µS/cm) | 0.16 |
| Soil Organic Matter | g kg^-1^ | 3.31 |
| Cation Exchange Capacity | mg kg^-1^ | 14.27 |
| N | mg kg^-1^ | 20.37 |
| P | mg kg^-1^ | 11.21 |
| K | mg kg^-1^ | 73.64 |
| S | mg kg^-1^ | 349.43 |
| Mo | mg kg^-1^ | 1.20 |

**Table S2:** Earthworm recovery at harvest.

| Treatment | Initial number (per pot) | Recovered number (per pot) | Survival rate  (%) |
| --- | --- | --- | --- |
| EW | 20 | 18 | 90 |
| n-MoS_2_ + EW (10 mg kg^-1^) | 20 | 16 | 80 |
